# Influencing the Activity of a Biocatalyst: The Combination of Temperature Selection and Substrate Properties Counts

**DOI:** 10.1021/acssuschemeng.5c07362

**Published:** 2025-10-16

**Authors:** Lena Graf, Klara M. Saller, Clemens Schwarzinger

**Affiliations:** Institute for Chemical Technology of Organic Materials, 27266 Johannes Kepler University Linz, Altenbergerstrasse 69, Linz 4040, Austria

**Keywords:** biocatalysis, polarity, acidity, bulk polymerization, polyester synthesis

## Abstract

Enzyme-catalyzed polyester synthesis is a promising alternative to conventional high-temperature melt polycondensations. Monomer properties significantly influence the performance of the enzyme catalyst, which depends on many other factors including temperature and immobilization technique. To understand the influences of substrate properties on the activity of *Candida antarctica* lipase B immobilized on Immobead 150, bulk reactions at 40, 60, or 80 °C were performed using adipic acid and aliphatic diols of differing lengths, where a constant N_2_ flow was employed for gentle water removal. Results from these isothermal reaction systems clearly indicate that the polarity of the diols and solubility of the acid in the reaction mixture have a major impact on the catalytic activity of the enzyme. While for the least polar long-chain diols (octanediol and decanediol) high conversions and molar masses were achieved at 80 °C, lower reaction temperatures were required for more polar diols to prevent catalyst inactivity (propanediol, butanediol, pentanediol, and hexanediol). For these substrates, temperature gradient programs were efficient in improving catalyst performance and increasing chain length. These findings highlight the importance of substrate evaluation and parameter screening, together with providing valuable insights for the application of biocatalysts in polyester synthesis.

## Graphical abstract

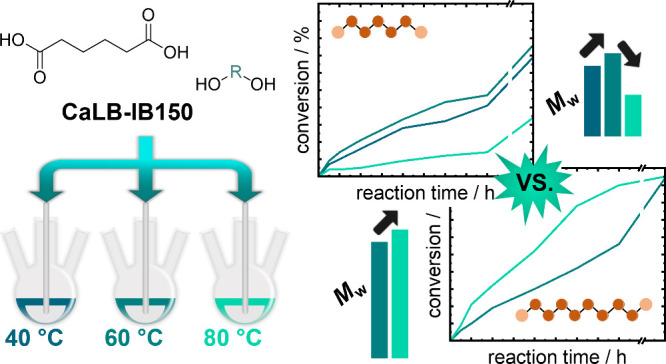

## Introduction

To achieve the goal of a greener polymer sector, the shift from fossil feedstock to biobased resources is commonly a topic of scientific discussion, polyesters being one of the most promising types of polymers.^1^ Considering the principles of green chemistry,^2,3^ the industrial synthesis of polyesters in melt has a potential for improvement concerning less hazardous synthesis routes, regarding the use of potentially toxic catalysts, diminishing the need for auxiliary substances such as entrainers and therefore preventing waste, and reduction in energy demands. Conventional syntheses require high reaction temperatures,^4^ and the condensation byproduct (e.g., water) is often removed using reduced pressure.^4^ In addition, monomers or resulting polymers can undergo thermal degradation, and side reactions are promoted due the high temperatures.^5^ Degradation^4,5^ or decarboxylation^6^ can occur, which leads to loss of functionality and discoloration of the product. Other side reactions include gelation due to cross-linking reactions,^1^ cyclization of oligomers and polymers,^7^ dehydration,^8^ or ether formation.^8^

Common catalysts used in polyester synthesis are metal oxides^9^ or organometal compounds,^9^ which are commonly not removed after production.^10^ The catalyst can migrate from the polymer during usage^10^ and accumulate in recycling processes^11^ or in nature after biodegradation of the polymer matrix.^12^ This poses risks as the catalyst remnants can be toxic.^13^ For example, antimony trioxide is used in the ppm range in the production of poly­(ethylene terephthalate)^14^ and has been found in bottled water^15^ and other food-contact materials.^16^ Furthermore, catalysts often lack selectivity, leading to difficulties in reactions of multifunctional monomers such as sugar-derived molecules.^8^

A different approach to conventional polyester synthesis involves the use of biocatalysts. Enzyme-catalyzed polyester synthesis has definite advantages compared to conventional polyester synthesis in terms of the principles of green chemistry.^2,3^ First of all, temperatures below 100 °C are already sufficient for enzymatic catalysts to achieve esterification,^17,18^ thus reducing energy expenses. Second, enzymes inherently exhibit stereo-,^19^ enantio-,^20^ regio-,^21^ or chemoselectivity,^21^ and the number of side reactions is reduced compared to nonenzymatic catalysts.^8^ In summary, enzymes as catalysts can be considered a greener option in polymer synthesis.

The selection of proper parameters for the synthesis of polyesters with enzymes is critical to achieving satisfactory activity of the biocatalyst. Polycondensation both in solvent^22,23^ as well as in bulk^8,18,22–28^ has been reported, but there is little information on how and why specific reaction parameters have been selected. Since additional components, such as solvents, should be avoided according to the principles of green chemistry,^2^ bulk polymerizations are preferred if at least one liquid monomer provides a homogeneous reaction mixture at selected temperatures.

Especially when it comes to reaction temperature, the information provided in the literature is often ambiguous. While the study of Korupp et al. reports a temperature of 60 °C as being beneficial in comparison to higher reaction temperatures in the synthesis of poly­(glyceryl adipate),^17^ Mahapatro et al. performed poly­(1,8-octylene adipate) synthesis at up to 90 °C with no significant changes to polymer growth.^18^ In a further study, Mahapatro et al. studied bulk polymerizations of aliphatic linear substrates with different chain lengths including succinic, glutaric, adipic, and sebacic acid and 1,4-butanediol, 1,6-hexanediol, and 1,8-octanediol. At the investigated reaction temperature of 70 °C, an increase of chain length of the diol in polymerizations with adipic acid resulted in an increase in the degree of polymerization. A similar trend when increasing the acid length in polymerizations with 1,8-octanediol was observed. The authors did not give any explanation for this phenomenon.^22^

Uyama et al. tested reaction temperatures of 50, 60, and 70 °C for sebacic acid and 1,4-butanediol,^28^ transferring the optimum 60 °C to a subsequent screening of diacids and diols with varying lengths.^23,28^ For adipic acid, these reaction conditions led to inferior yields with ethylene glycol and 1,4-butanediol as reaction partners, although very high catalyst concentrations of approximately 25% w/w were used. The study by Campisano et al. investigated diacids, including adipic acid, in polymerizations with diols at 90 °C. In the case of short-chain monomers, the molar mass increase was mainly attributed to autocatalysis since no significant differences were observed between enzyme-catalyzed and uncatalyzed reference experiments. The hydrophobicity of substrates was pointed out as one possible factor influencing the chain-length preference of the enzymatic catalyst.^25^ Binns et al. investigated the polymerization of adipic acid with 1,4-butanediol^24^ or 1,6-hexanediol,^24,26^ and concluded that the solubility of the diacid is higher in short-chain diols, leading to an increased acidity in the reaction medium and partial enzyme deactivation.

Our recent study^29^ focused on the synthesis of poly­(butylene adipate) using *Candida antarctica* lipase B immobilized on Immobead 150 (CaLB-IB150). Using a comparably low catalyst concentration of 1% w/w required careful optimization of reaction conditions to avoid inactivity of the enzyme. We started to combine and extend existing theories about the role of acidity and polarity on the performance of the catalyst in polycondensation reactions and how these factors are influenced by temperature. To further investigate our previous hypothesis that enzyme inactivity is mainly caused by the polarity- and temperature-dependent acidity of the reaction medium, a broader screening including linear diols of varying chain lengths and different temperatures is presented in this study. The systematic identification of suitable reaction conditions promotes the applicability of enzyme catalysis in technologically relevant bulk polycondensations.

## Materials and Methods

### Materials

Adipic acid (99%, Thermo Scientific), 1,3-propanediol (≥99%, Thermo Scientific), 1,4-butanediol (≥99%, Carl Roth), 1,5-pentanediol (98%, Fisher Scientific), 1,6-hexanediol (≥96%, Carl Roth), 1,8-octanediol (98%, Apollo Scientific), and 1,10-decanediol (99%, Thermo Scientific) were used as supplied. The catalyst *Candida antarctica* lipase B, immobilized on Immobead 150, recombinant from *Aspergillus oryzae*, was purchased from Sigma-Aldrich. For analysis, dimethyl sulfoxide-*d*_6_ (99.8%, Deutero), chloroform-*d*_1_ (99.8% + Ag, Deutero), unstabilized tetrahydrofuran (≥99.9%, Roth), and polystyrene standards with molar masses of 162, 945, 3090, 6660, 12,980, 27,060, 67,600 (Agilent Technologies), 90,000, and 206,000 g mol^–1^ (Pressure Chemical) were employed as received from the suppliers.

### General Procedure for Enzyme-Catalyzed Bulk Polycondensations

Bulk polycondensations (Scheme 1) were performed in a three-neck round-bottom flask (250 mL), which was equipped with a mechanical stirrer and heated by an oil bath. In all reactions, adipic acid was used as the diacid, and the molar ratio between diacid and diol was 1:1.05 to achieve hydroxy terminated polyesters. Monomer quantities were adjusted to give 40 g polyester. For the liquid diols (up to 1,5-pentanediol), adipic acid, the corresponding diol, and the immobilized enzyme (0.4 g, 1% w/w in regard to polyester synthesized) were added directly to the reaction flask and heated, defined as the start of the reaction. In the case of 1,6-hexanediol, 1,8-octanediol, and 1,10-decanediol, first the diol was allowed to melt in the reaction flask, and the subsequent addition of adipic acid and the catalyst was defined as the starting time. For all reactions, 0.1 L min^–1^ N_2_ flow was applied for water removal, and isothermal conditions of 40, 60, or 80 °C were investigated. Samples were taken after 0.5, 1, 2, 4, 6, 8, and 24 h. Further experiments using an increase of temperature from 40 to 60 °C for 1,3-propanediol and 1,4-butanediol or from 60 to 80 °C for 1,6-hexanediol with additional pressure reduction to 20 mbar after 8 h were performed. The two-step synthesis was run for 72 h with an additional sample taken after 48 h. Samples were stored at −30 °C to ensure the inactivity of the enzyme catalyst and to prevent further reaction. A complete list of experiments is given in Table S2 in the Supporting Information (SI).

**Scheme 1:**
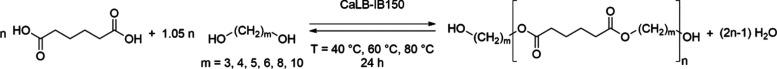
General Reaction Scheme for the Enzyme-Catalyzed Polyester Synthesis Reactions Using Adipic Acid and Different Diols at Varying Reaction Temperatures

### Solubility Tests of Adipic Acid in Linear Diols

Two hundred milligrams of the respective diols was weighed with increasing amounts of adipic acid (±1 mg) in individual experiments. The mixtures were stirred at different temperatures for 1 h and checked to see whether a clear solution was obtained. Thus, a resolution of 0.005 g g^–1^ adipic acid in diol was achieved.

$$\mathrm{conversion}\;(\mathrm{DMSO})/\%=\frac{A_{2.35-2.25\mathrm{ppm}}}{A_{2.35-2.25\mathrm{ppm}}+A_{2.25-2.15\mathrm{ppm}}}\times 100$$ 1

$$\mathrm{conversion}\;({\mathrm{CDCl}}_{3})/\%=\frac{A_{4.11-3.98\mathrm{ppm}}}{A_{4.11-3.98\mathrm{ppm}}+A_{3.69-3.58\mathrm{ppm}}}\times 1.05\times 100$$ 2

### Determination of Conversion Based on ^1^H Nuclear Magnetic Resonance Spectroscopy

Samples were dissolved in dimethyl sulfoxide-*d*_6_ or chloroform-*d*_1_, depending on their solubility, at approximately 25 mg mL^–1^, and ^1^H nuclear magnetic resonance (^1^H NMR) spectra were recorded on a Bruker Avance 300 MHz. Peaks are given in ppm relative to tetramethylsilane and were calibrated using the solvent signal. In dimethyl sulfoxide-*d*_6_, peaks for methylene groups adjacent to the carboxylic acid groups of adipic acid shifted from 2.25–2.15 to 2.35–2.25 ppm upon esterification. This allowed the calculation of conversion using peak areas *A* according to eq 1. Since no shift was observed for adipic acid in CDCl_3_, which was needed to dissolve polyesters with longer diols, the methylene groups of the diols adjacent to the hydroxyl (3.69–3.58 ppm) and ester (4.11–3.98 ppm) groups were used instead (eq 2).

### Size Exclusion Chromatography

Size exclusion chromatography was performed using an Agilent Series 1200 setup with a degasser, binary pump, autosampler, column compartment, and diode array detector connected to a Shodex RI-71 refractive index detector. The analytes were separated using a Phenogel precolumn (30 × 4.6 mm, 5 μm, linear) and three Phenogel columns (300 × 4.6 mm, 5 μm) with pore sizes of 50, 500, and 10,000 Å at 40 °C using a flow rate of 0.350 mL min^–1^. Samples were analyzed at a concentration of 1 mg mL^–1^ in unstabilized tetrahydrofuran. Molar masses (*M*_n_, *M*_w_) and dispersity (*Đ*) were determined by using the refractive index detector and conventional calibration with polystyrene standards. Peaks were cut off at the elution time of the smallest standard (162 g mol^–1^), thus excluding the monomers 1,6-hexanediol, 1,5-pentanediol, 1,4-butanediol, and 1,3-propanediol (Figure S10 in the Supporting Information). Samples were analyzed without any workup.

## Results and Discussion

### Influence of Temperature and Substrate Properties in Bulk Polycondensations

To evaluate the effect of substrate properties on the catalytic activity of *Candida antarctica* lipase B immobilized on Immobead 150 in correlation with reaction temperature, aliphatic diols of different lengths were tested in combination with adipic acid. To investigate these parameters selectively, reaction systems were kept simple. Single temperatures of 40, 60, and 80 °C were applied over 24 h, while gentle removal of reaction water was achieved by a stream of N_2_ over the reaction mixture.

To compare the polarity of the monomers, log*P* values^30^ or the Hildebrand solubility parameters^31^ have been described in the literature. While the former is the logarithm of the partition coefficient between octanol and water,^32^ the latter involves the cohesive energy density of a liquid.^33^ For this study, Hansen solubility parameters were chosen, which are based on the Hildebrand solubility parameter but divide it further into dispersion forces (nonpolar) and polar forces and H-bonding.^33^ Therefore, Hansen solubility parameters allow for a clearer evaluation of the involved properties of substances such as polarity. When comparing the polarity of the diols according to the data provided by the “Hansen Solubility Parameters in Practice” (HSPiP) software,^34^ a clear trend between the chain length of the diol and its polarity is visible (Figure 1). The presence of adipic acid in the reaction mixture also adds to the absolute value of these polarities (adipic acid: δ_P_ = 10.0 MPa^1/2^) but does not change the trend.^34^

**Figure 1:**
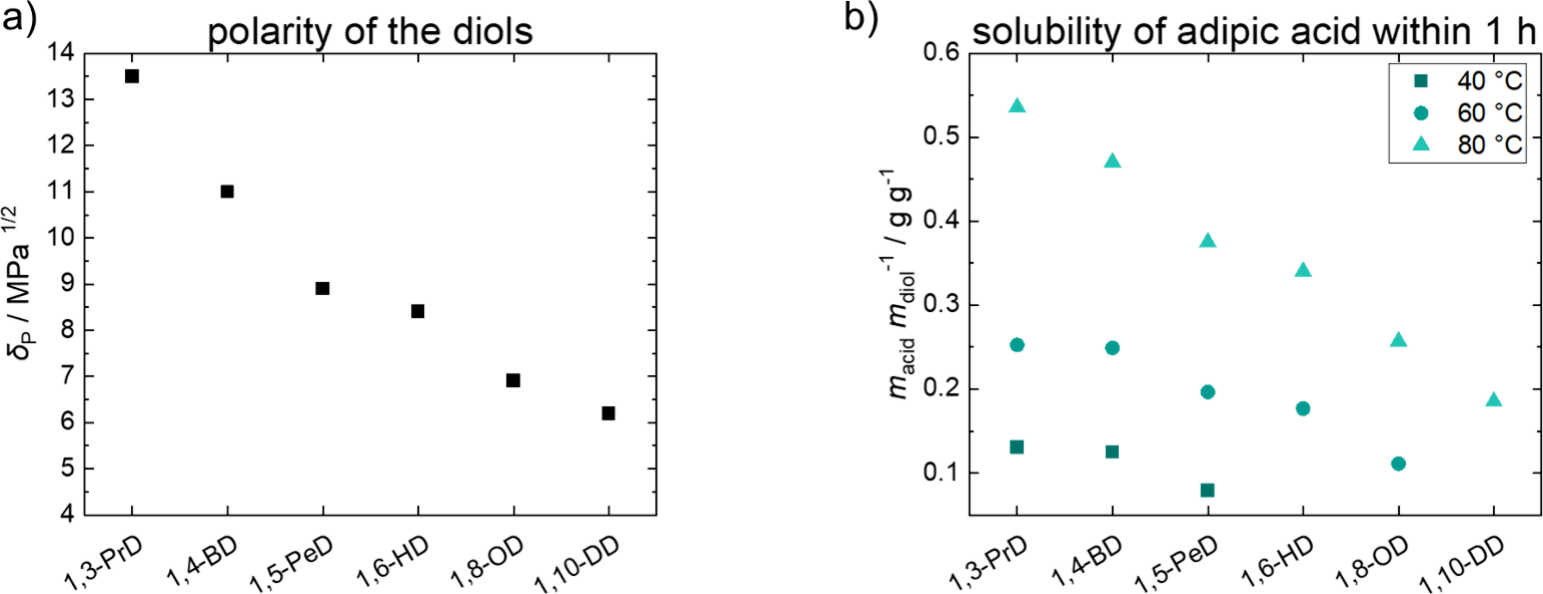
(a) Polarity of diols according to the Hansen solubility parameters^34^ and (b) solubility of adipic acid in the respective diols 1,3-propanediol (1,3-PrD), 1,4-butanediol (1,4-BD), 1,5-pentanediol (1,5-PeD), 1,6-hexanediol (1,6-HD), 1,8-octanediol (1,8-OD), and 1,10-decanediol (1,10-DD).

Another factor is the acidity of the reaction mixture introduced by adipic acid (p*K*_a1_ = 4.41, p*K*_a2_ = 5.11 in water at 30 °C).^35^ The influence of the acid on the properties of the reaction mixture and therefore also its influence on the biocatalyst are a function of the acid’s solubility in the reaction mixture.^29^ The solubility of adipic acid in the respective diol (melt) was determined at 40, 60, and 80 °C (Figure 1). A time frame of 1 h was chosen based on the observation in bulk polymerizations that while the acid was not completely dissolved at the start of the reaction, the reaction mixtures turned clear with increasing conversion. This indicated the formation of oligomers and an improved solubility of the acid in the reaction mixture compared to the diol melt present in the beginning of the reaction.

As presented in Figure 1, the solubility of adipic acid increased with decreasing chain length of the diol, the trend well aligning with respective polarities. Further, solubility significantly increased at higher temperatures. Rising temperature is additionally known to lower dissociation constants and thus increase the acidity of aqueous media.^35^ Extrapolating the findings on the dependence of p*K*_a1_ values for adipic acid on the dielectric constant of the medium allowed for a rough estimation of the concentration of dissociated adipic acid in the liquid diols (C_3_–C_5_) at 40 and 60 °C.

Although the solubility of adipic acid in 1,5-pentanediol at 60 °C is higher than that in 1,4-butanediol at 40 °C, the difference in p*K*_a1_ values led to comparable results for the carboxylate concentrations in these two mixtures. This concentration nearly doubles when increasing the temperature for butanediol to 60 °C due the effects of both solubility and p*K*_a1_ value. Details on p*K*_*a*_ calculations are given in SI (Section 7). The influence of these effects on the biocatalyst was studied in polycondensation reactions of adipic acid in different diols at varying temperatures.

When investigating conversions of adipic acid with 1,3-propanediol, 1,4-butanediol, and 1,5-pentanediol at 40 °C (Figure 2), the reaction rate was similar for all diols up to 8 h reaction time, with final conversions of 65–85% within 24 h. Furthermore, conversion rates of all three diols showed a steeper slope during the first 2 h of reaction but a slowing down until 8 h. This is also the time span where more advanced reaction schemes have been reported to provide a change in parameters, such as increase in temperature or lowering reaction pressure for enhanced byproduct removal.^24,26,27,29^ Although the reaction using 1,4-butanediol resulted in higher molar masses (*M*_w_ of 1960 g mol^–1^), correlating to the highest conversion, molar masses overall remained comparably low after 24 h (*M*_w_ around 1200 g mol^–1^ for 1,3-propanediol and 1,5-pentanediol) (Figure 3). Representative ^1^H NMR spectra of the reactions with different monomer combinations are given in the Supporting Information (Figures S1–S9). As bulk polymerizations were performed, the melting temperatures of the diols represent the lowest possible reaction temperatures.

**Figure 2:**
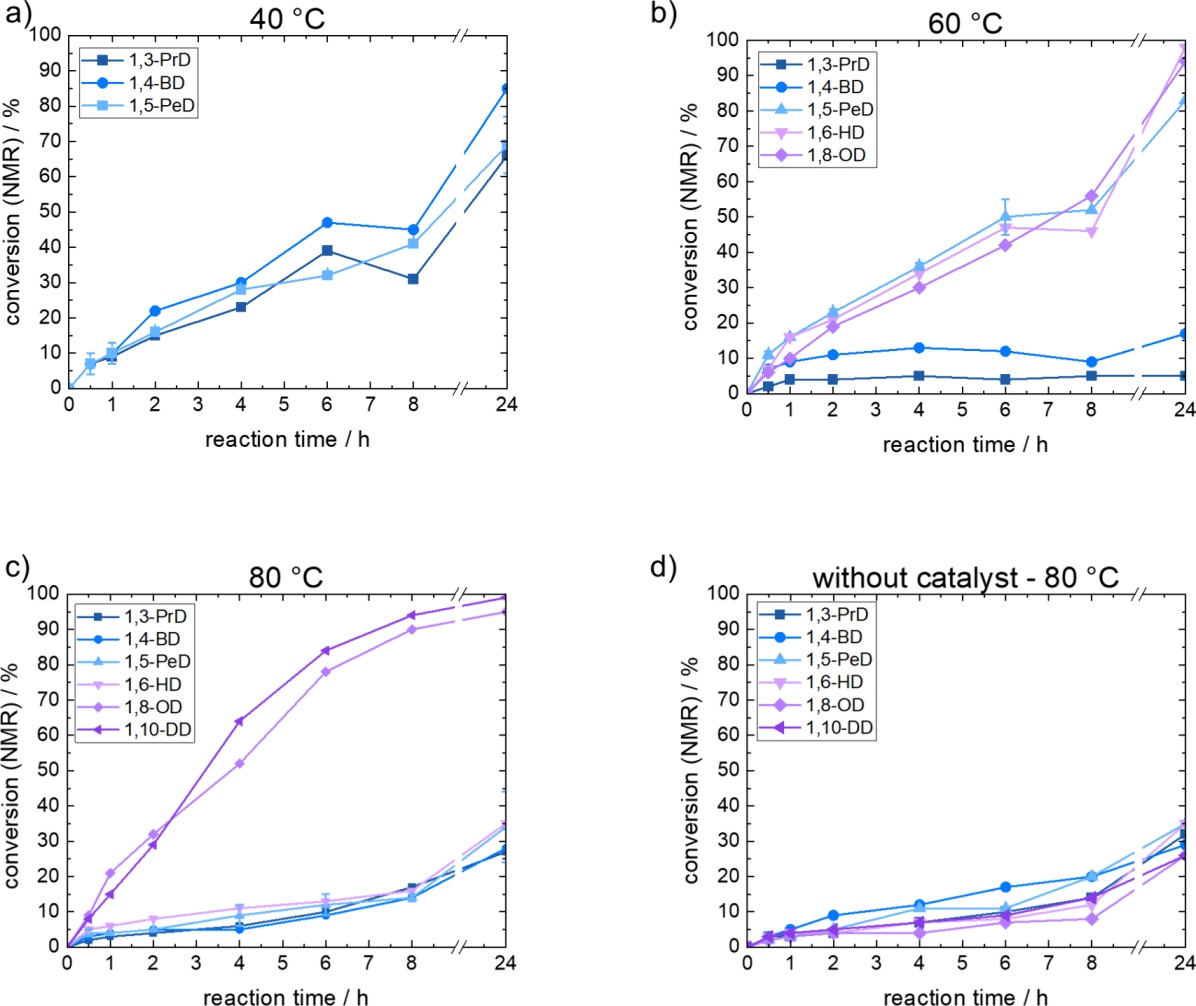
Conversion of adipic acid during 24 h reaction time using 1,3-propanediol (1,3-PrD), 1,4-butanediol (1,4-BD), 1,5-pentanediol (1,5-PeD), 1,6-hexanediol (1,6-HD), 1,8-octanediol (1,8-OD), and 1,10-decanediol (1,10-DD) at (a) 40 °C, (b) 60 °C, or c­() 80 °C reaction temperature, including (d) conversions of reactions without a catalyst at 80 °C.

**Figure 3:**
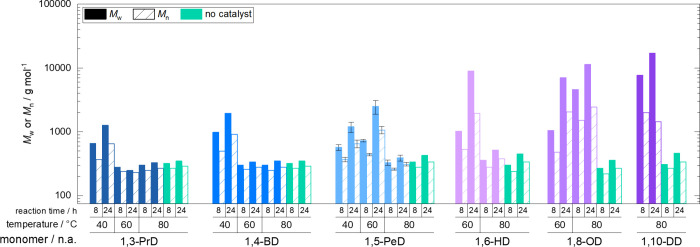
Molar masses after 8 and 24 h reaction time using 1,3-propanediol (1,3-PrD), 1,4-butanediol (1,4-BD), 1,5-pentanediol (1,5-PeD), 1,6-hexanediol (1,6-HD), 1,8-octanediol (1,8-OD), and 1,10-decanediol (1,10-DD) at 40, 60, or 80 °C reaction temperature and reactions at 80 °C without a catalyst (*M*_w_ as filled columns and *M*_n_ as crosshatched columns).

When changing the reaction temperature from 40 to 60 °C (Figure 2), conversions dropped for the two most polar diols, i.e., 1,3-propanediol and 1,4-butanediol, while remaining in a similar range as the 40 °C experiments for the longer diols. Final conversions of close to 100% were achieved for 1,6-hexanediol and 1,8-octanediol at 60 °C. These trends were also reflected in increased molar masses for 1,6-hexanediol and 1,8-octanediol after 24 h (*M*_w_ = 8930 and 7060 g mol^–1^). 1,5-Pentanediol reached an *M*_w_ of 2500 g mol^–1^, whereas 1,3-propanediol and 1,4-butanediol stayed at around 300 g mol^–1^ (Figure 3). 1,5-Pentanediol was selected for reproducibility studies, and the isothermal reactions were repeated in duplicate at 40, 60, and 80 °C. As can be seen in Figures 2 and 3, satisfactory reproducibility was achieved; details are given in Table S4 in the SI.

A similar trend in behavior was observed when the temperature was raised from 60 to 80 °C (Figure 2). Now, diols up to a chain length of six carbons showed only minimal reaction, while 1,8-octanediol and 1,10-decanediol gave fast, rather linear reaction rates for the first 6 h (with conversions of roughly 80%). Chloroform-*d*_1_ was used as a solvent for ^1^H NMR analysis due to enhanced solubility of polyesters from long-chain diols. Only in the sample taken after 4 h was residual adipic acid not dissolved completely, and conversion is thus slightly overestimated. After 6 h, clear solutions in CDCl_3_ and final turnovers of 95 and 100% were obtained. This is reflected by the molar masses of the final products including 1,8-octanediol (*M*_w_ = 11,370 g mol^–1^) and 1,10-decanediol (*M*_w_ = 16,990 g mol^–1^).

All the shorter diols showed only a very slow reaction and finally reached a conversion of 30% after 24 h. Control experiments showed that all of the diols, regardless of their chain length, behaved exactly the same when no enzyme was added to the reaction. This proved that the esterification was due to thermal autocatalytic polycondensation (Figure 2). According to the literature, the phenomenon of temperature-induced esterification seems to be dependent on temperature and the exact substrates used.^18,25,28^

Although the literature commonly correlates the preference of the lipase for longer substrates to hydrophobic interactions^25,36^ with the architecture of the catalytic pocket,^37^ we found distinct correlations between our polycondensation results (Figures 2 and 3), the polarity of the diols, and the solubility of adipic acid (Figure 1). The shorter and therefore more polar the diols are, the lower the reaction temperature must be for the enzyme to stay catalytically active. We address this behavior to the amount of adipic acid and, therefore, the acidity present in the reaction mixture. With increasing diol length, the acid becomes less soluble, and thus, higher temperatures can be used while still not deactivating the enzyme.

Our results allow for the reevaluation of previous studies on enzyme-catalyzed polyester synthesis with linear substrates and observed chain length preferences. Uyama et al. found an optimum reaction temperature of 60 °C for the reaction of sebacic acid and 1,4-butanediol, which only gave very low yields for adipic acid with short-chain diols.^23,28^ The generalization of reaction parameters seems to be hindered by different substrate properties like p*K*_a_ values for the acids, sebacic acid (p*K*_a1_ = 4.72, p*K*_a2_ = 5.45)^38^ and adipic acid (p*K*_a1_ = 4.41, p*K*_a2_ = 5.11),^35^ together with their polarities (adipic acid: δ_P_ = 10.0 MPa^1/2^, sebacic acid: δ_P_ = 7.1 MPa^1/2^).^34^ Similar inferior conversions for adipic acid reported by Mahapatro et al.^22^ and Campisano et al.^25^ could also be explained by the combination of acidity, polarity, and resulting temperature sensitivity of *Candida antarctica* lipase B.

Another aspect to be considered is the formation of water as a byproduct. Water removal will shift the equilibrium of the esterification reaction in favor of polyester formation, but a certain water content has been found to be beneficial for the enzyme-catalyzed polymerization of ε-caprolactone.^39^ While screening experiments were carried out under different isobaric conditions (atmospheric^23,25,28,29^ or under reduced pressure^22^), the application of byproduct removal by a vacuum after a certain reaction time^24,26,27,29^ has been shown to have beneficial effects on the polymer chain length.

### Influence of Advanced Reaction Parameters on the Results of Bulk Polycondensations

As previously mentioned, isothermal reaction systems were selected to understand the influence of polarity and acidity in correlation with the temperature. For enhancing conversion and molar masses, more advanced reaction schemes were investigated. While in the beginning of the reaction lower temperatures and nitrogen flow for gentle water removal were applied, the temperature was increased after 8 h, while pressure was decreased to 20 mbar to remove water from the reaction mixture.

To show that a temperature increase during the reaction is beneficial, experiments with three diols were performed. 1,3-Propanediol and 1,4-butanediol were selected for a temperature increase from 40 to 60 °C after 8 h as these two diols caused a decline in catalyst activity under isothermal conditions when temperatures of 60 °C were directly used (Figure 2). 1,6-Hexanediol was selected for investigation of a reaction system showing the same behavior at 80 °C (Figure 2). The reaction time of 8 h at which parameter variation was performed was based on the slowing down of the reaction rate and our previous investigation.^29^ At this point, the conversion of carboxylic acid groups reached 40–50%.

Using the temperature increase and pressure reduction after 8 h resulted in a significant improvement of conversions to about 90 to 100% after 24 h (Figure 4) compared to 30% and less if the reactions were directly carried out at the higher temperatures. This was accompanied by increased molar masses of the polymers (Figure 5). When the chromatograms of the three reactions were compared, it was visible that the dispersities of the samples taken after 8 h showed clear differences (Figure S11, Supporting Information). While dispersity is 1.6 for reactions with 1,3-propanediol and 1,4-butanediol, dispersity reached 3.2 for the reaction with 1,6-hexanediol. In addition, clear signals of different oligomers were visible as has been observed previously in other studies.^24,26^ ^,^ ^40^ After 24 and 48 h, molar masses significantly increased, as did dispersities. The latter can be explained by the fact that no workup of the polymer has been performed as described in the literature^18^ and all oligomeric species were included in the molar mass calculation. A complete list of molar mass values and dispersities is given in the SI (Table S3, Supporting Information).

**Figure 4:**
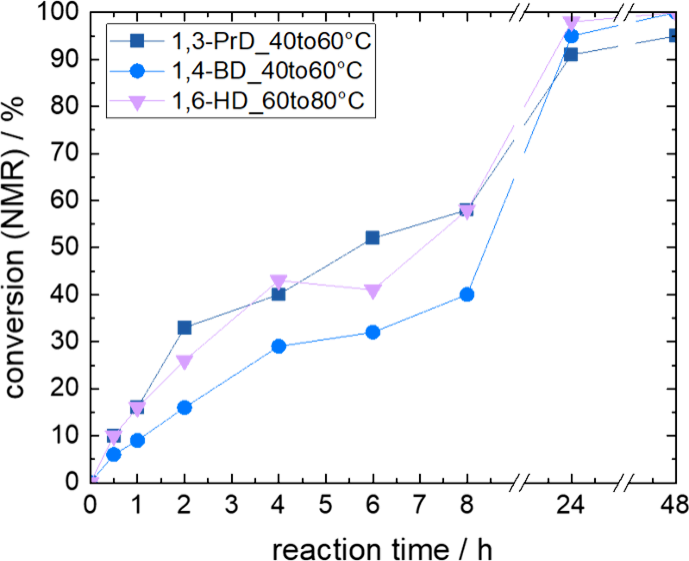
Conversion of adipic acid of reactions with 1,3-propanediol (1,3-PrD), 1,4-butanediol (1,4-BD), and 1,6-hexanediol (1,6-HD) during 48 h reaction time, where temperatures were increased from 40 to 60 °C for 1,3-PrD and 1,4-BD or from 60 to 80 °C for 1,6-HD after 8 h reaction time, while for all reactions, pressure was reduced to 20 mbar after 8 h.

**Figure 5:**
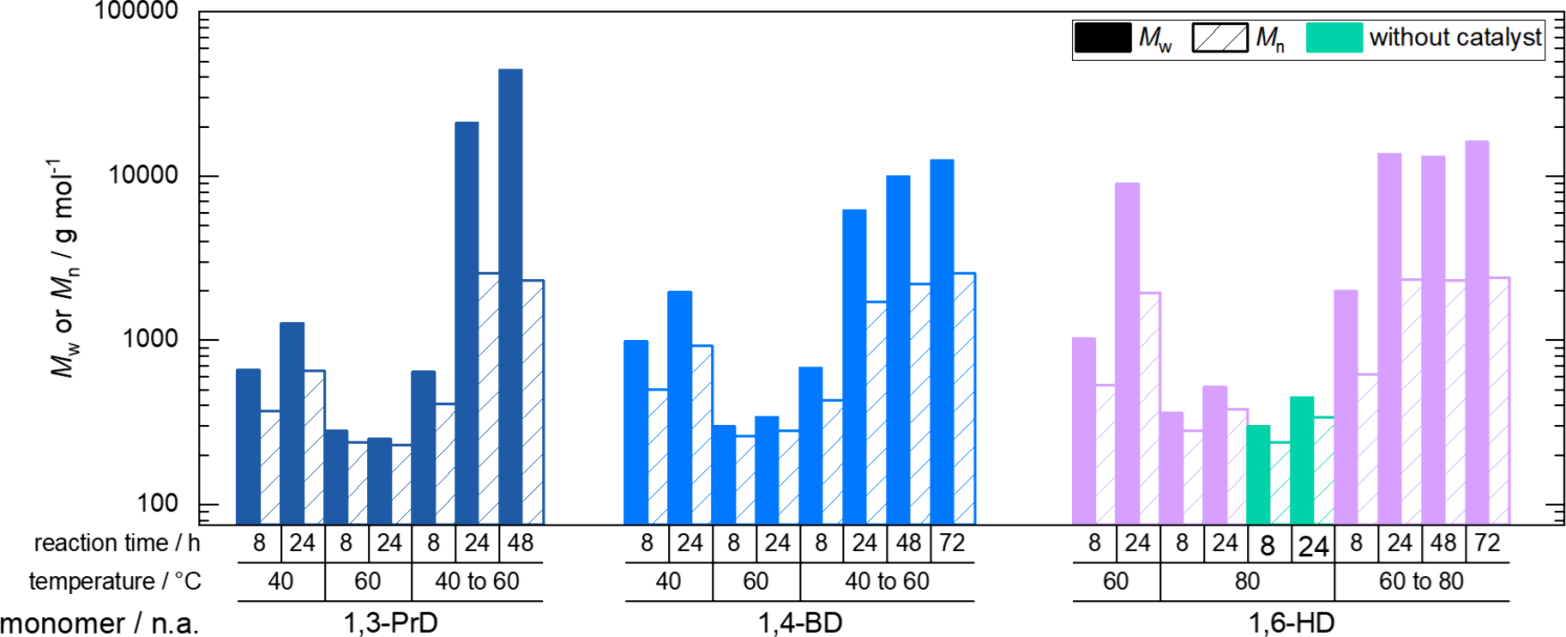
Comparison of molar masses obtained from isothermal and advanced reaction conditions after 8, 24, 48, and 72 h reaction time using 1,3-propanediol (1,3-PrD), 1,4-butanediol (1,4-BD), and 1,6-hexanediol (1,6-HD) (*M*_w_ as filled columns and *M*_n_ as crosshatched columns).

The increased conversion and molar masses proved the positive effect of the oligomerization step at lower temperatures. This could be explained by different conditions in the enzyme′s environment in the beginning of the reaction compared to an oligomerization phase. Upon oligomerization, the acidity and polarity of the reaction mixture change as, on the one hand, less free acid is available upon oligomer formation. On the other hand, polarity declines as fewer unbound monomers are present. To visualize the reduction of polarity of monomers in comparison with oligomers, polarities were estimated using the “Hansen Solubility Parameters in Practice” (HSPiP) software.^34^ Even though small differences in polarity between estimated and experimental values provided by the software were observed (Table S1, Supporting Information), the polarity significantly changed upon the formation of oligomers as visualized in Figure 6. In addition, polarities depended on the kind of end groups of oligoesters, although differences reduced with chain length and reached similar values among all monomer systems.

**Figure 6:**
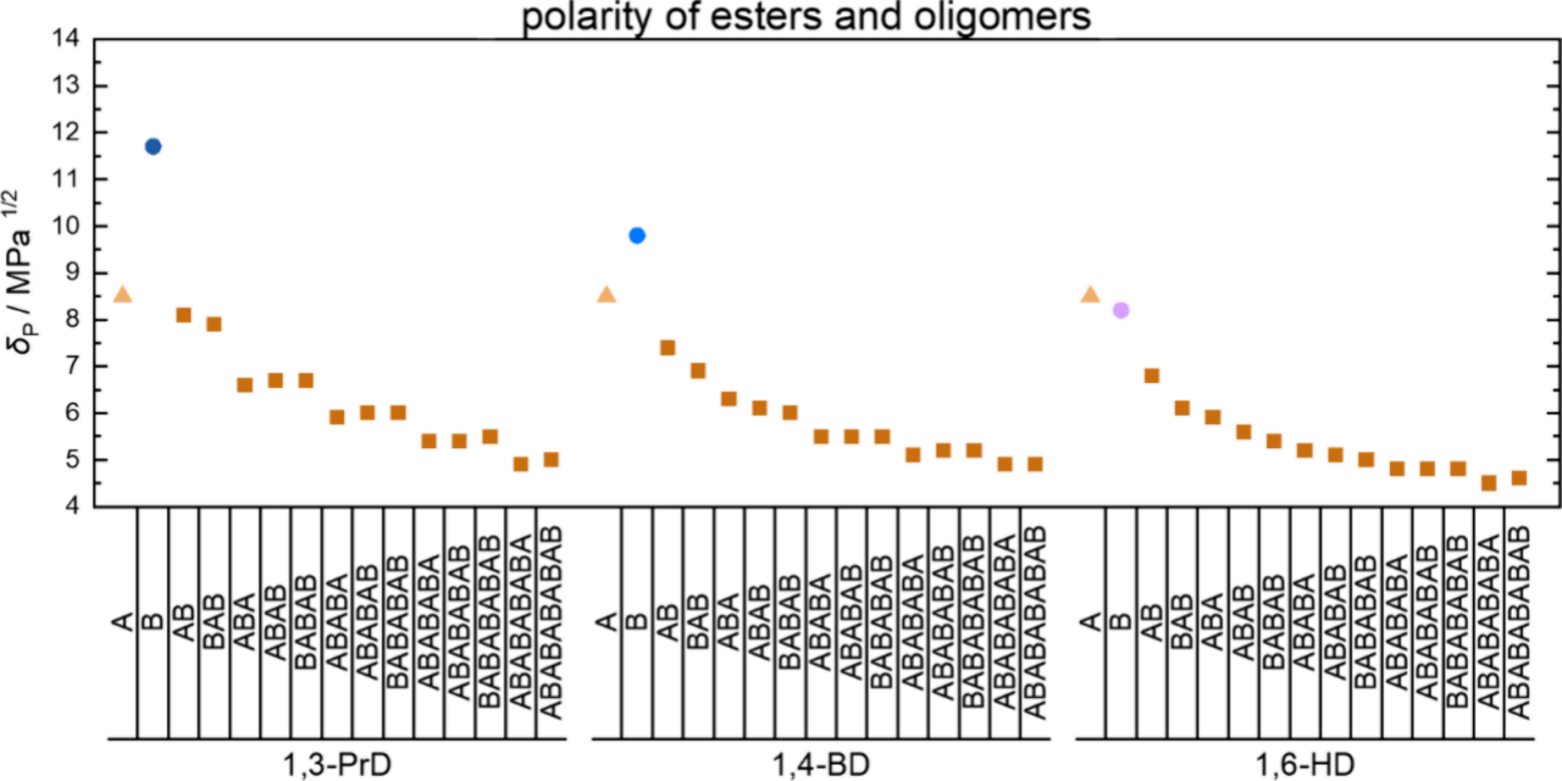
Polarities of adipic acid (A, triangles); diols (B, circles) including 1,3-propanediol (1,3-PrD), 1,4-butanediol (1,4-BD), and 1,6-hexanediol (1,6-HD); and the respective oligoesters with varying lengths and end groups estimated using HSPiP.^34^

Thus, the higher the conversion of monomers is, the less free diol and the more oligomers of lower polarity are present in the reaction mixture. In turn, the polarity of the reaction mixture and therefore solubility of the acids are reduced to a value that allows for higher reaction temperatures. The higher reaction temperature combined with the improved water removal due to the reduction in pressure thus led to a significantly increased conversion and molar masses, which would otherwise be impossible with isothermal temperature schemes.

## Conclusions

This study demonstrates the significant impact of temperature, polarity, solubility of the acid, and therefore acidity of the reaction mixture on the outcome of a polyester synthesis using biocatalysts such as *Candida antarctica* lipase B immobilized on Immobead 150. It was shown that the solubility of adipic acid in different diols is influenced by temperature and diol chain length. While for 1,3-propanediol and 1,4-butanediol a starting temperature of 40 °C was needed, 1,5-pentanediol and 1,6-hexanediol required a temperature of 60 °C, and significant conversions for 1,8-octanediol and 1,10-decanediol were reached at 80 °C. It was shown that the polarity of the reaction mixture is lowered with increasing degree of polymerization leading to a lower concentration of free acid, which allows an increase of reaction temperature over time. By applying advanced reaction schemes, it was possible to achieve excellent conversions for all tested monomers within 24 h. Our results indicate a critical need in parameter screening according to the substrate to be tested and that caution should be taken for generalization of suitable substrates as parameters employed can lead to misrepresentations. This study aids in future evaluation of monomers and provides insight in how substrate parameters can play a vital role in the success of a polyester synthesis using biocatalysts.

## Supplementary Material



## Notes

Complete list of experiments including molar masses of reaction products and investigation of reproducibility, ^1^H NMR spectra, size exclusion chromatograms, further data regarding polarities of the monomers, and estimation of pKa values (PDF)

## Abbreviations

1,3-PrD: 1,3-propanediol
1,4-BD: 1,4-butanediol
1,5-PeD: 1,5-pentanediol
1,6-HD: 1,6-hexanediol
1,8-OD: 1,8-octanediol
1,10-DD: 1,10-decanediol
ADPA: adipic acid
CaLB-IB150: *Candida antarctica* lipase B immobilized on Immobead 150
^1^H NMR: ^1^H nuclear magnetic resonance
