# Supplementary material for: Influencing the Activity of a Biocatalyst: The Combination of Temperature Selection and Substrate Properties Counts
Source: ACS Sustain Chem Eng. 2025 Oct 16;13(42):18094–101. doi: 10.1021/acssuschemeng.5c07362 (PMC12570358; doi:10.1021/acssuschemeng.5c07362)
Supplement: Supplementary file 1 [file sc5c07362_si_001.pdf]

Supplementary information

Influencing the activity of a biocatalyst – the combination of temperature selection and substrate properties counts

Lena Graf, Klara M. Saller*, Clemens Schwarzinger

Institute for Chemical Technology of Organic Materials, Johannes Kepler University Linz, Austria

Number of pages: 16

Number of figures: 13

Number of tables: 5

Contents

1. NMR spectra S2
2. Size exclusion chromatograms S7
3. Polarity of monomers according to HSPiP S8
4. List of polycondensation experiments. S9
5. Molar mass of reaction products S10
6. Investigation of reproducibility S13
7. Estimation of pKa values S15

1. NMR spectra.

^{1}H-NMR spectra are given for reactions with different diols in Figures S1–S9. While the labelling of protons in respective diols is explained in each individual graph, reaction products with varying reaction partners are given as superscripts. Thus, A1 refers to adipic acid monomer, $A1^{Pr}$ describes adipic acid which reacted with one propanediol unit (monoester, terminating group), and $A1^{PrPr}$ describes products after the reaction with two diol units (diester). The monoester has $A1^{Pr}$ groups neighboring either the carboxylic acid or the ester group, causing signals overlapping with both the monomer and the diester peak. In case of adipic acid, the shift of A1 protons can be used for calculating the conversion in DMSO-d_6.

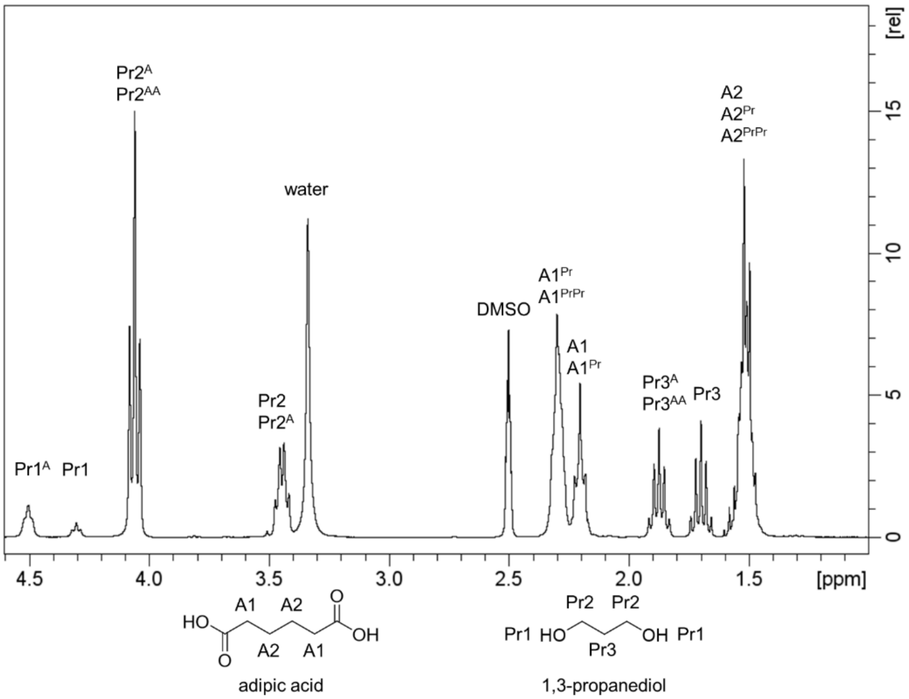

FIGURE S1: ^{1}H-NMR spectrum of a reaction using adipic acid and 1,3-propanediol in dimethyl sulfoxide-d_6 (reaction number 1 after 24 h reaction time in Table S2).

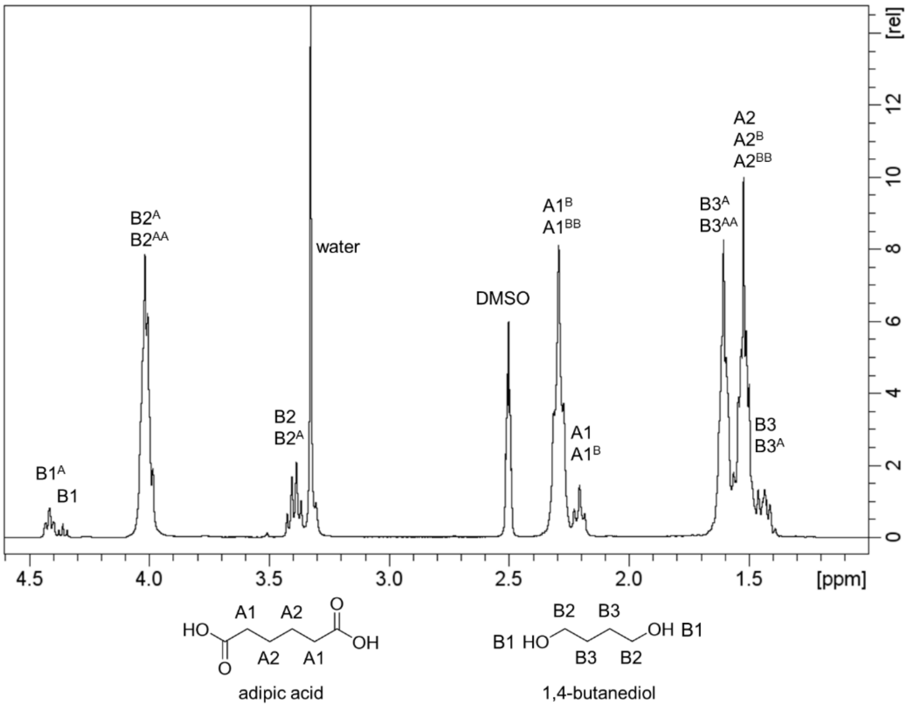

FIGURE S2: ^{1}H-NMR spectrum of a reaction using adipic acid and 1,4-butanediol in dimethyl sulfoxide-d_6 (reaction number 6 after 24 h reaction time in Table S2).

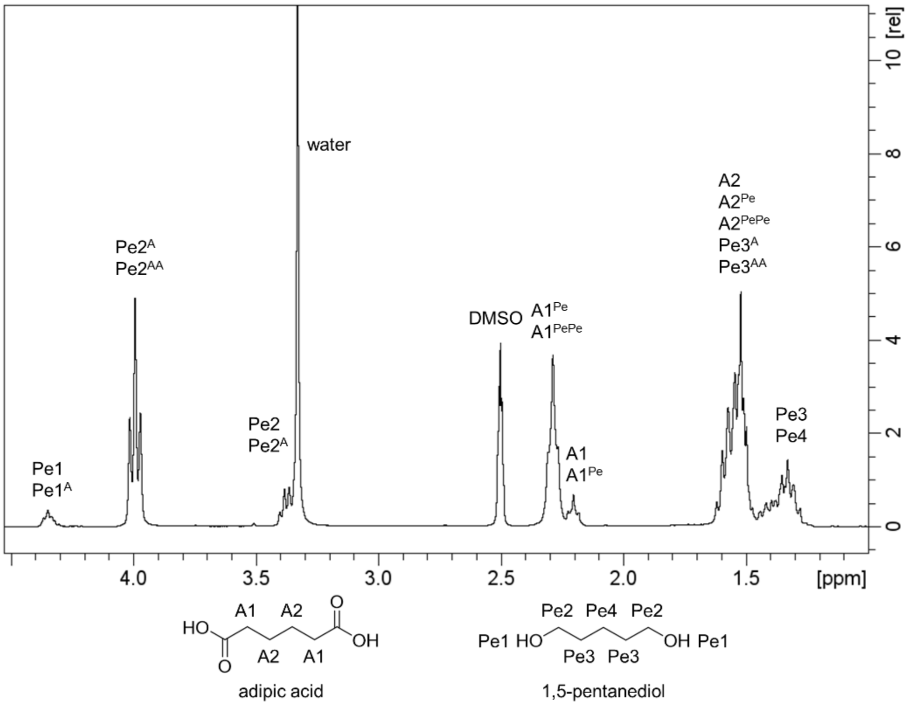

FIGURE S3: ^{1}H-NMR spectrum of a reaction using adipic acid and 1,5-pentanediol in dimethyl sulfoxide-d_6 (reaction number 13 after 24 h reaction time in Table S2).

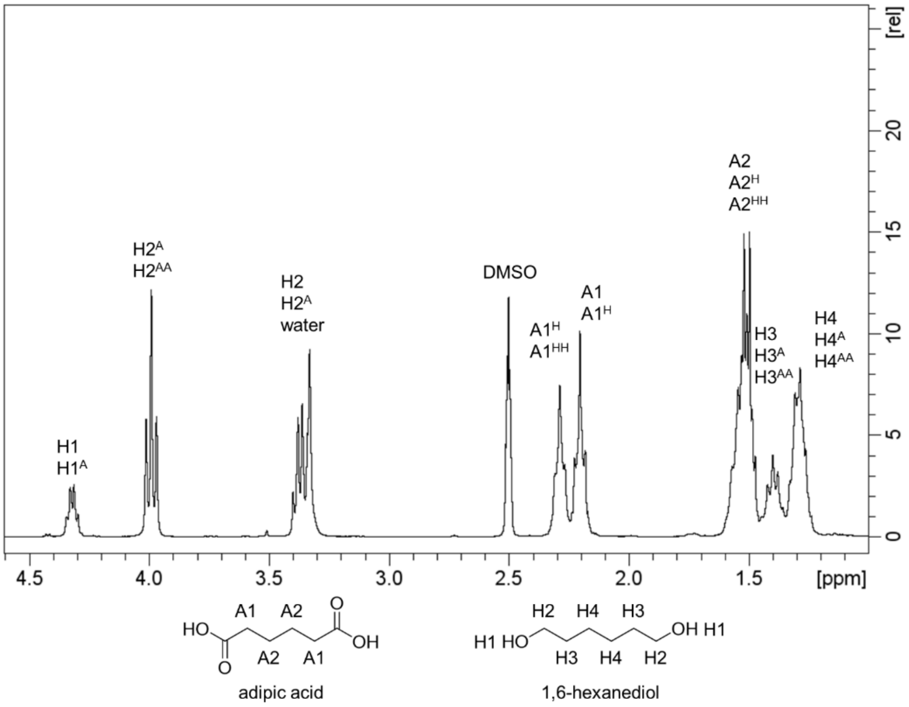

FIGURE S4: ^{1}H-NMR spectrum of a reaction using adipic acid and 1,6-hexanediol in dimethyl sulfoxide-d_6 (reaction number 18 after 8 h reaction time in Table S2).

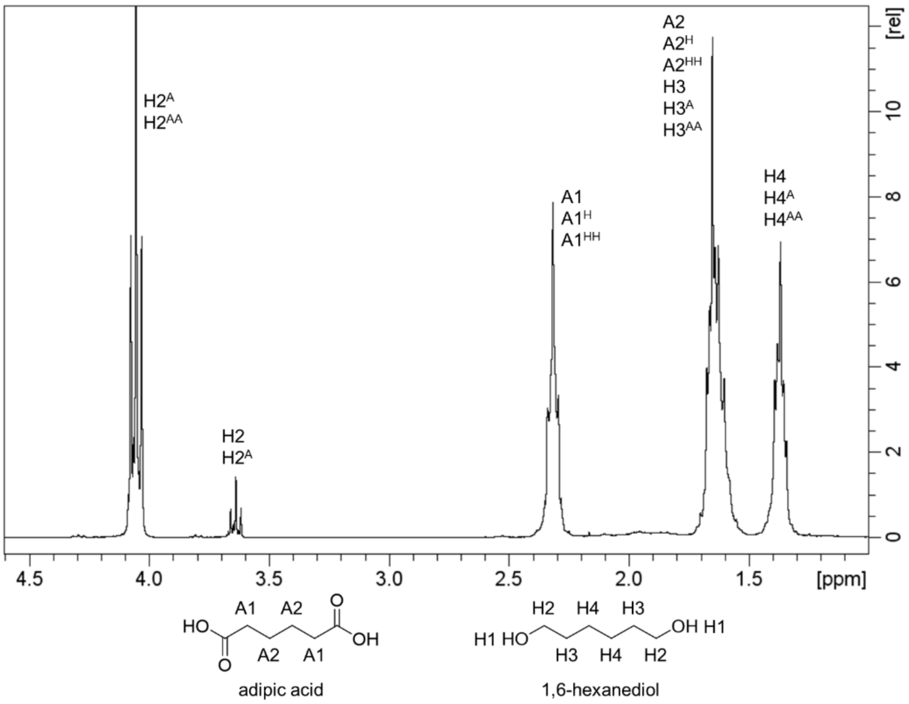

FIGURE S5: ^{1}H-NMR spectrum of a reaction using adipic acid and 1,6-hexanediol in chloroform-d_1 (reaction number 18 after 24 h reaction time in Table S2).

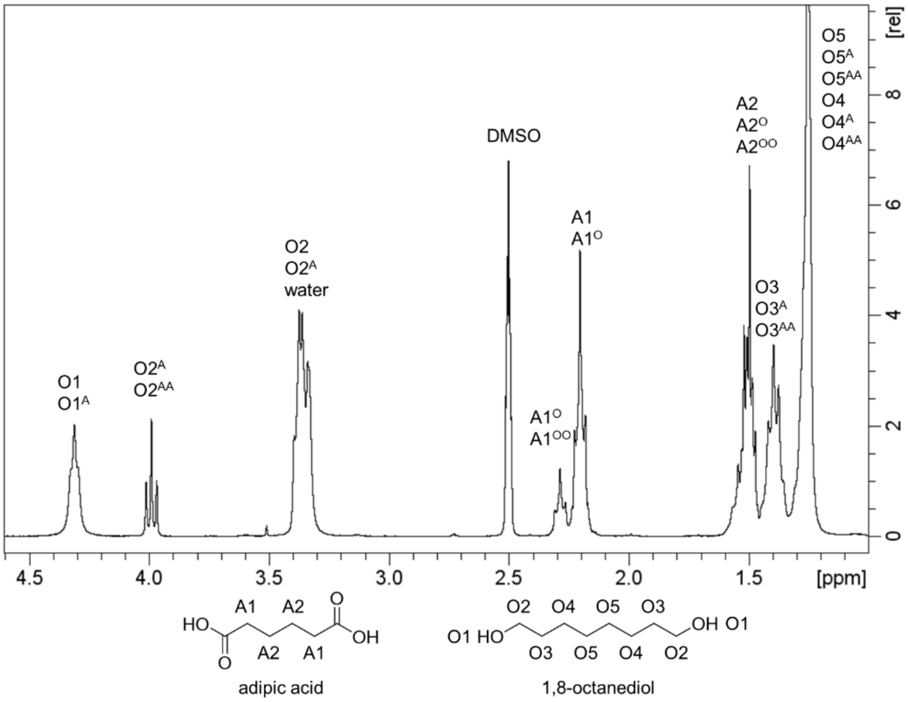

FIGURE S6: ^{1}H-NMR spectrum of a reaction using adipic acid and 1,8-octanediol in dimethyl sulfoxide-d_6 (reaction number 23 after 1 h reaction time in Table S2).

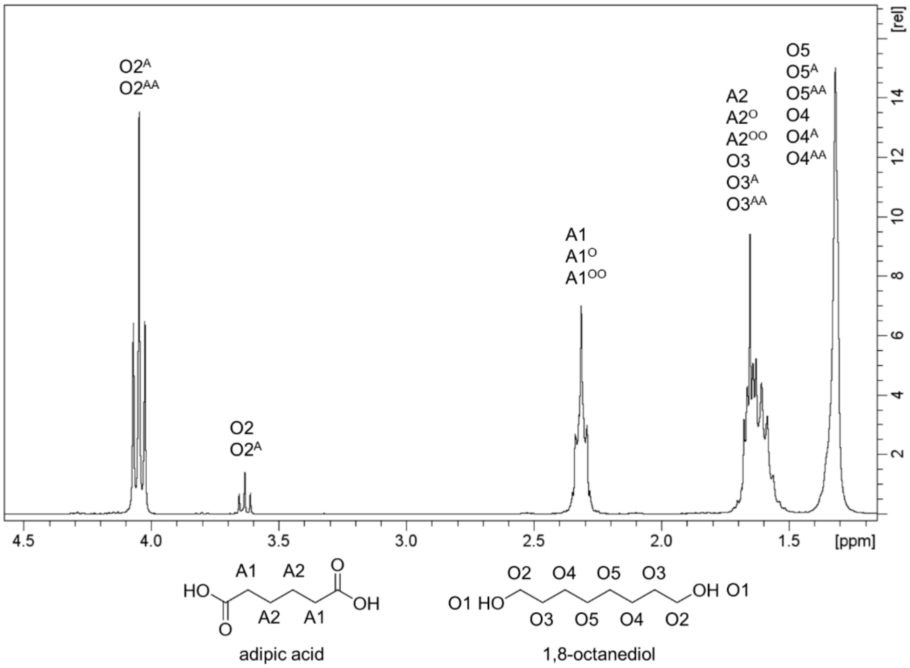

FIGURE S7: ^{1}H-NMR spectrum of a reaction using adipic acid and 1,8-octanediol in chloroform-d_1 (reaction number 23 after 24 h reaction time in Table S2).

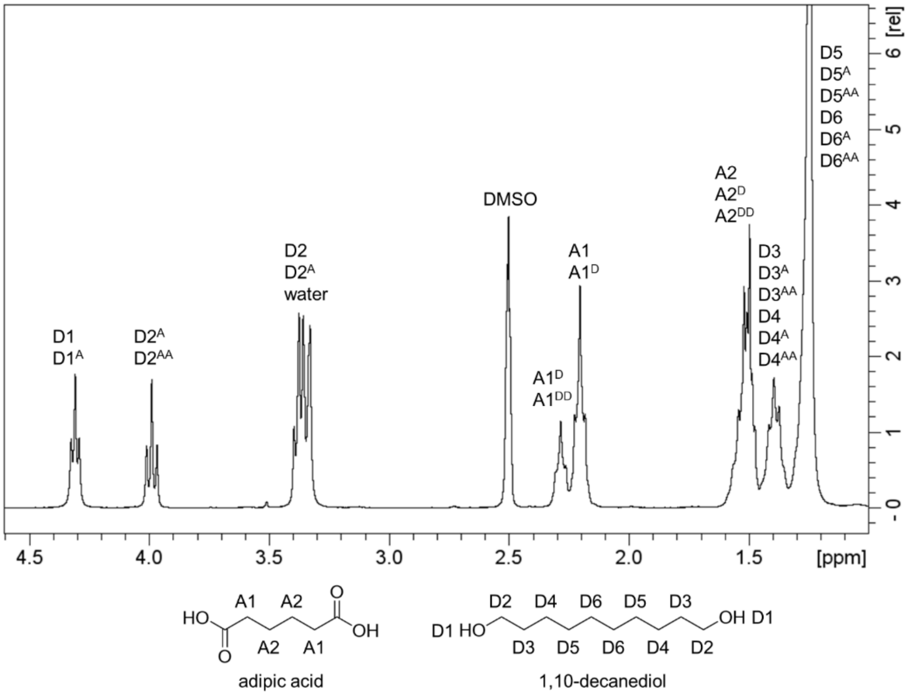

FIGURE S8: ^{1}H-NMR spectrum of a reaction using adipic acid and 1,10-decanediol in dimethyl sulfoxide-d_6 (reaction number 25 after 2 h reaction time in Table S2).

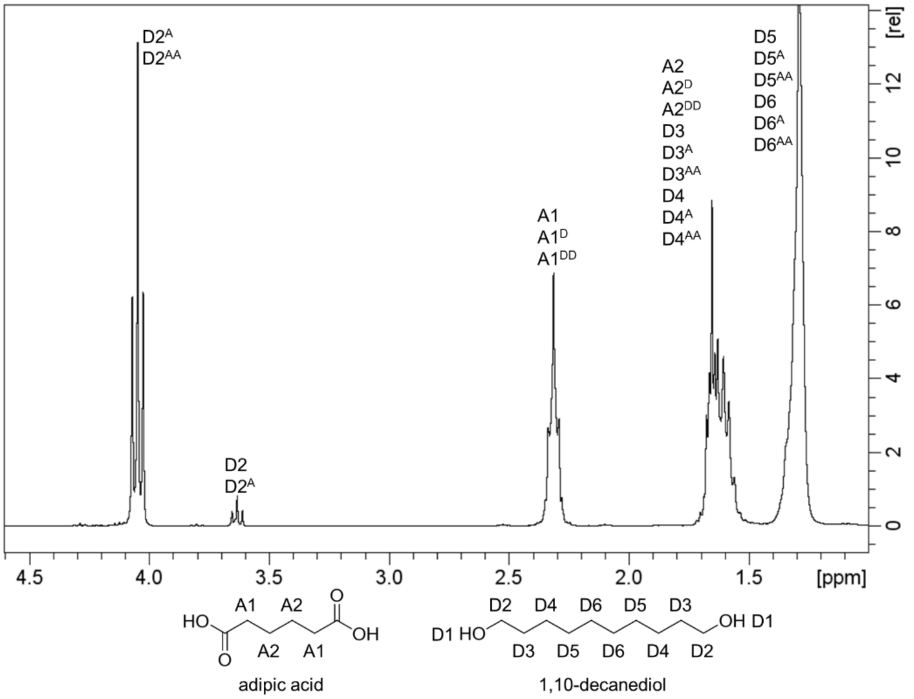

FIGURE S9: ^{1}H-NMR spectrum of a reaction using adipic acid and 1,10-decanediol in chloroform-d_1 (reaction number 25 after 24 h reaction time in Table S2).

2. Size exclusion chromatograms.

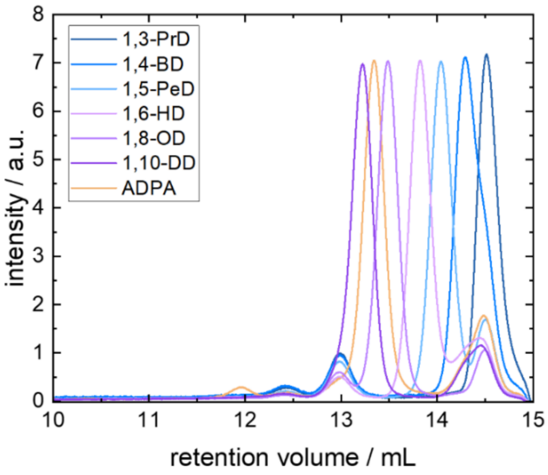

FIGURE S10: Results of size exclusion chromatography of monomers 1,3-propanediol (1,3-PrD), 1,4-butanediol (1,4-BD), 1,5-pentanediol (1,5-PeD), 1,6-hexanediol (1,6-HD), 1,8-octanediol (1,8-OD), 1,10-decanediol (1,10-DD) and adipic acid (ADPA).

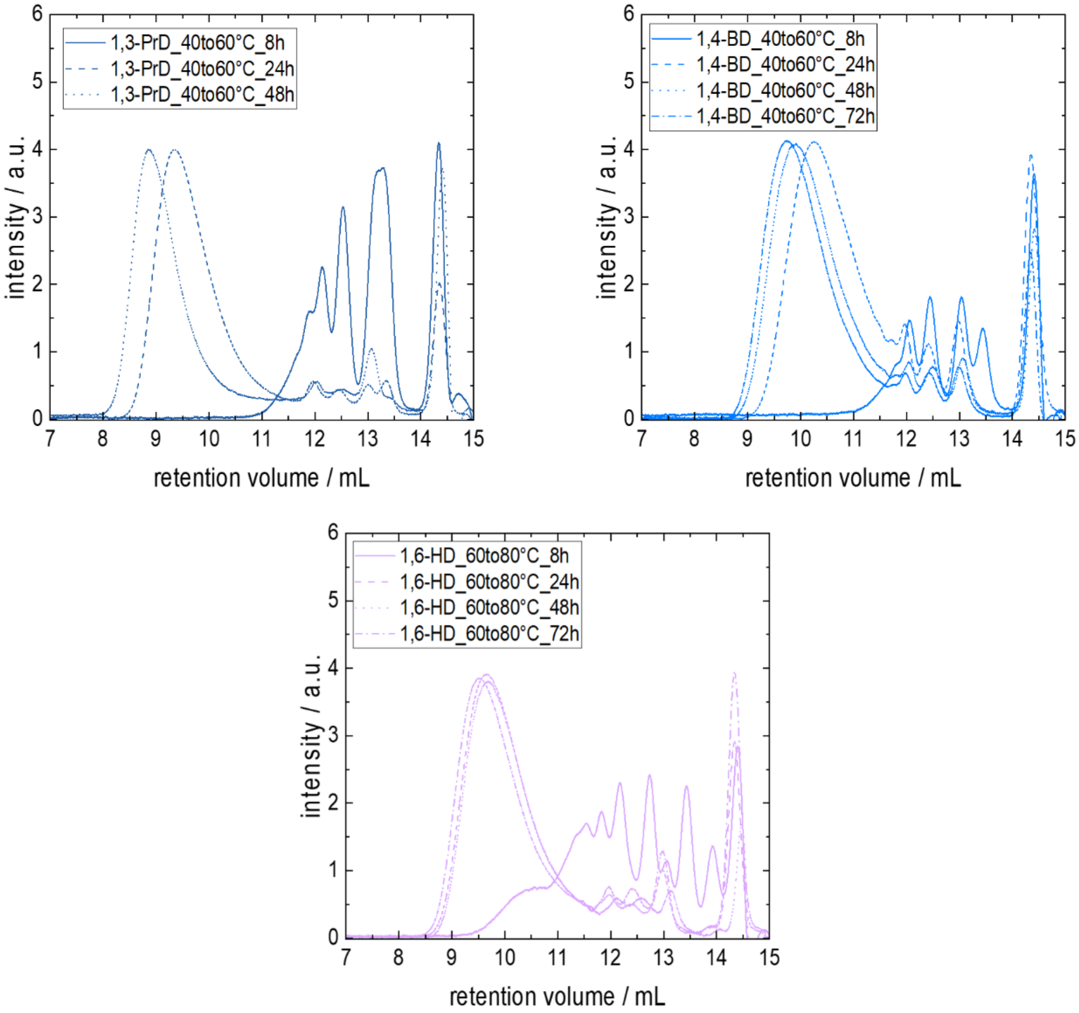

FIGURE S11: Results of size exclusion chromatography of two-step reactions using adipic acid, and 1,3-propanediol (1,3-PrD), 1,4-butanediol (1,4-BD) and 1,6-hexanediol (1,6-HD).

3. Polarity of monomers according to HSPiP.

TABLE S1: Polarity of monomers according to HSPiP.

monomer	δ_P (estimated) / MPa$^{1/2}$	δ_P (database) / MPa$^{1/2}$
adipic acid	8.5	10.0
1,3-propanediol	11.7	13.5
1,4-butanediol	9.8	11.0
1,6-hexanediol	8.2	8.4

4. List of polycondensation experiments.

TABLE S2: Parameters of the reactions with adipic acid.

reaction number	diol	temperature / °C	water removal
1	1,3-propanediol	40	N_2
2	1,3-propanediol	60	N_2
3	1,3-propanediol	80	N_2
4*	1,3-propanediol	80	N_2
5	1,3-propanediol	8 h 40°C, 16 h 60 °C	8 h N_2, 16 h 20 mbar
6	1,4-butanediol	40	N_2
7	1,4-butanediol	60	N_2
8	1,4-butanediol	80	N_2
9*	1,4-butanediol	80	N_2
10	1,4-butanediol	8 h 40°C, 16 h 60 °C	8 h N_2, 16 h 20 mbar
11	1,5-pentanediol	40	N_2
12	1,5-pentanediol	40	N_2
13	1,5-pentanediol	60	N_2
14	1,5-pentanediol	60	N_2
15	1,5-pentanediol	80	N_2
16	1,5-pentanediol	80	N_2
17*	1,5-pentanediol	80	N_2
18	1,6-hexanediol	60	N_2
19	1,6-hexanediol	80	N_2
20*	1,6-hexanediol	80	N_2
21	1,6-hexanediol	8 h 60°C, 16 h 80 °C	8 h N_2, 16 h 20 mbar
22	1,8-octanediol	60	N_2
23	1,8-octanediol	80	N_2
24*	1,8-octanediol	80	N_2
25	1,10-decanediol	80	N_2
26*	1,10-decanediol	80	N_2

*reaction without biocatalyst

5. Molar mass of reaction products.

TABLE S3: Molar mass of reaction products.

reaction number	reaction time / h	M_n / g mol-1	M_w / g mol-1	$Đ$
1	8	370	660	1.8
	24	650	1270	2.0
2	8	240	280	1.2
	24	230	250	1.1
3	8	250	300	1.2
	24	270	330	1.2
4*	8	270	320	1.2
	24	290	350	1.2
5	8	410	640	1.6
	24	2560	21030	8.2
	48	2320	44380	19.1
6	8	500	980	2.0
	24	920	1960	2.1
7	8	260	300	1.2
	24	280	340	1.2
8	8	250	300	1.2
	24	280	350	1.2
9*	8	270	320	1.2
	24	290	350	1.2
10	8	430	680	1.6
	24	1720	6160	3.6
	48	2190	9980	4.6
	72	2560	12500	4.9
11	8	340	510	1.5
	24	560	980	1.8

*reaction without biocatalyst

TABLE S3 (continued): Molar mass of reaction products.

reaction number	reaction time / h	M_n / g mol-1	M_w / g mol-1	*Đ*
12	8	400	630	1.6
	24	730	1420	2
13	8	450	760	1.7
	24	1210	3110	2.6
14	8	420	700	1.6
	24	930	1890	2
15	8	270	350	1.3
	24	320	420	1.3
16	8	250	300	1.2
	24	290	350	1.2
17*	8	280	340	1.2
	24	340	430	1.3
18	8	530	1020	1.9
	24	1950	8930	4.6
19	8	280	360	1.3
	24	380	520	1.4
20*	8	240	300	1.2
	24	340	450	1.3
21	8	620	1990	3.2
	24	2340	13650	5.8
	48	2310	13130	5.7
	72	2400	16110	6.7
22	8	480	1050	2.2
	24	2050	7060	3.5
23	8	1510	4600	3
	24	2450	11370	4.6

*reaction without biocatalyst

TABLE S3 (continued): Molar mass of reaction products.

reaction number	reaction time / h	M_n / g mol-1	M_w / g mol-1	$Đ$
24*	8	220	270	1.2
	24	270	360	1.4
25	8	2000	7660	3.8
	24	1450	16990	11.8
26*	8	270	310	1.2
	24	340	460	1.4

*reaction without biocatalyst

6. Investigation of reproducibility.

TABLE S4: Investigation of reproducibility.

reaction number	reaction time / h	conversion (NMR) / %	M_n / g mol-1	M_w / g mol-1	$Đ$
1+5	0.5	8±1	-	-	-
	1	13±3	-	-	-
	2	24±9	-	-	-
	4	32±9	-	-	-
	6	46±6	-	-	-
	8	44±14	390±20	650±10	1.7±0.1
6+10	0.5	6±1	-	-	-
	1	9±0	-	-	-
	2	19±3	-	-	-
	4	29±0	-	-	-
	6	39±7	-	-	-
	8	42±3	470±40	830±150	1.8±0.2
11+12	0.5	7±3	-	-	-
	1	10±3	-	-	-
	2	16±1	-	-	-
	4	28±1	-	-	-
	6	32±1	-	-	-
	8	41±1	370±30	570±60	1.6±0.1
	24	69±8	650±90	1200±220	1.9±0.1

TABLE S4 (continued): Investigation of reproducibility.

reaction number	reaction time / h	conversion (NMR) / %	M_n / g mol-1	M_w / g mol-1	$Đ$
13+14	0.5	11±1	-	-	-
	1	16±0	-	-	-
	2	23±1	-	-	-
	4	36±1	-	-	-
	6	50±5	-	-	-
	8	52±0	440±20	730±30	1.7±0
	24	83±0	1070±140	2500±610	2.3±0.3
15+16	0.5	4±1	-	-	-
	1	4±0	-	-	-
	2	5±0	-	-	-
	4	9±3	-	-	-
	6	12±3	-	-	-
	8	14±1	260±10	330±30	1.3±0.1
	24	34±10	310±20	390e±40	1.3±0.1
18+21	0.5	8±1	-	-	-
	1	16±0	-	-	-
	2	23±3	-	-	-
	4	39±4	-	-	-
	6	44±3	-	-	-
	8	51±5	575±50	1505±490	3±0.7

7. Estimation of pKa values.

The concentration of dissociated adipic acid in liquid diols was estimated based on the dependence of the pK_{a1} value on the dielectric constant ε which itself depends on temperature [G.A. El-Naggar; *Talanta* 47 (1998) 1013–1020.]. Relevant experimental data presented in El-Naggar's publication have been plotted in Figures S12 and S13. The temperature-dependency of ε has been extrapolated from presented data to 1,3-propanediol (1,3-PrD), 1,4-butanediol (1,4-BD), and 1,5-pentanediol (1,5-PeD) which are liquid at room temperature and for which dielectric constants are reported [CRC Handbook of Chemistry and Physics, CRC Press, Boca Raton, FL, 2005]. After extrapolation of $1/\varepsilon$, a linear relation was used to calculate pK_{a1}. Using the experimental solubility of adipic acid as described in the manuscript allowed for the estimated concentration of dissociated adipic acid. Results are summarized in Table S5.

TABLE S5: Calculated $1/\varepsilon$, pK_{a1}, and concentration of dissociated adipic acid in different liquid diols at varying temperatures.

	**40 °C**			**60 °C**		
Diol	$1/\varepsilon$	pK_{a1}	c (COO^-) / mmol L^{-1}	$1/\varepsilon$	pK_{a1}	c (COO^-) / mmol L^{-1}
1,3-Propanediol	0.0323	5.44	4.3	0.0362	5.25	7.3
1,4-Butanediol	0.0344	5.56	3.5	0.0386	5.37	6.2
1,5-Pentanediol	0.0435	6.09	1.5	0.0488	5.88	3.0

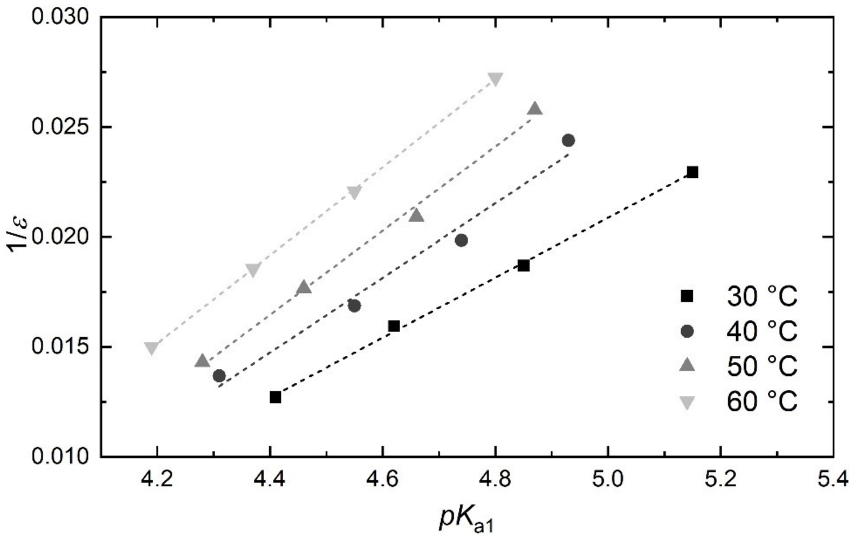

FIGURE S12: Linear correlation of pKa1 and $1/\varepsilon$ at different temperatures as reported by El-Naggar.

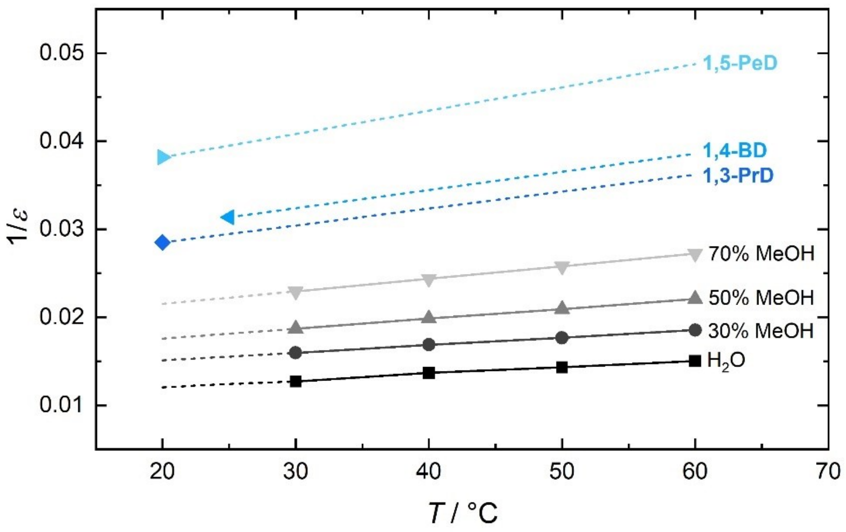

FIGURE S13: Extrapolated 1/ε values (dashed lines) at different temperatures for liquid diols 1,3-propanediol (1,3-PrD), 1,4-butanediol (1,4-BD), and 1,5-pentanediol (1,5-PeD) following the trend of water and water/methanol (MeOH) mixtures given by El-Naggar.
